# Delayed vascular complication after collagenase injection for Dupuytren disease

**DOI:** 10.1186/s12891-023-06964-z

**Published:** 2023-10-23

**Authors:** Jesper Nordenskjöld, Jonas Nilsson, Roua Kalaf, Isam Atroshi

**Affiliations:** 1 Department of Orthopedics, Hässleholm- Kristianstad Hospitals, Hässleholm, Sweden; 2 Department of Clinical Sciences- Orthopedics, Lund University, Lund, Sweden; 3 Department of Radiology, Kristianstad Hospital, Kristianstad, Sweden

**Keywords:** Dupuytren´s contracture, Collagenase, Adverse events, Vascular complication

## Abstract

**Background:**

Vascular adverse events after collagenase injection for Dupuytren disease are absent in large trials and systematic reviews. The aim of this study is to present a case series of delayed vascular complications after collagenase treatment.

**Methods:**

A prospective evaluation of 1181 consecutively treated patients at one orthopedic department identified three patients reporting symptoms of possible vascular complication. Baseline demographics and description of symptoms were collected, with a physical examination documenting extension deficit and neurovascular status. All patients completed the Cold Intolerance Symptom Severity (CISS) scale (range 4-100, lower is better) and underwent Doppler sonography examination of the digital arteries.

**Results:**

All patients were treated in the small finger and two had an isolated proximal interphalangeal joint contracture. All patients had a delayed presentation of a few months, with episodes of white discoloration of the treated finger relieved within 30 min and associated with variable pain, paresthesia, stiffness and weakness. Two of the patients reported cold exposure as an episode trigger and had a pathological CISS score (40 and 36, respectively). Doppler sonography identified a nonpatent ulnar digital artery in one patient.

**Conclusions:**

Delayed vascular complication after collagenase treatment is rare, but surgeons and patients should be aware of the risk, especially when treating the small finger.

## Background

In recent years minimally invasive treatment methods for Dupuytren disease, such as needle fasciotomy and collagenase injection, have gained in popularity due to rapid recovery and low rate of severe adverse events [1, 2]. A severe and well-described adverse event after surgical fasciectomy is neurovascular injury [3]. Collagenase, consisting of class I and II isoforms, disrupts Dupuytren cords by exerting a catalytic effect on all types of collagen except type IV, which is the main component in basement membranes of neurovascular structures [4]. Preclinical studies have demonstrated preservation of arterioles and nerves following local injection [5]. Furthermore, in large trials and systematic reviews, no neurovascular injuries are reported after collagenase treatment [6, 7, 8, 9].

Spiers et al. presented a case report in 2014 of a patient presenting with a delayed persistent vascular complication two weeks following collagenase treatment for Dupuytren disease manifesting with episodes of bluish discoloration of the treated small finger [10]. Doppler sonography and an arteriogram revealed a nonpatent ulnar digital artery distal to the proximal interphalangeal (PIP) joint. Kawano et al. reported a similar case in 2019 of a patient with delayed onset of Raynauds phenomenon episodes after treatment of the small finger [11]. The specific cause and prevalence of these reported vascular complications after collagenase treatment are unknown.

In this study we present a case series of patients with a delayed vascular complication after treatment with collagenase injection for Dupuytren disease.

## Methods

We prospectively evaluated all patients treated with collagenase injection for Dupuytren disease at one orthopedic department from November 2011 through June 2020. The department is the only center that treats Dupuytren disease in a region with approximately 300,000 inhabitants, and no patients are referred for treatment at other centers.

During the study period, collagenase injection was the first-line treatment choice for primary Dupuytren disease at the department. Treatment indication consisted of a palpable Dupuytren cord and an active extension deficit (AED) of at least 20° in the metacarpophalangeal (MCP) and/or the PIP joint. A single hand surgeon treated all patients using a modified method, injecting a higher dose (approximately 0.80 mg) into multiple spots in the cord [12]. The injection was followed by an extension procedure after one or two days. Local anesthesia (buffered mepivacaine) was administered as a nerve block in the proximal palm before both the injection and the extension procedure [13]. After the extension procedure, a hand therapist applied a static splint to be used during nighttime for 8 weeks.

Patients were followed up at 6 weeks and at 2, 3 or 5 years. All possible adverse events were registered.

### Patients with possible vascular complication

Patients who reported symptoms related to a possible vascular complication at any time during the study period were asked to participate in this study. All included patients received oral and written information and provided written consent, and this report follows the STROBE guidelines. Baseline data (including sex, age, smoking status, comorbidities, and heredity for Dupuytren disease) and description of symptoms were obtained. Baseline and posttreatment AED of the treated finger joints and the occurrence of skin tears recorded during their initial follow-up examinations were retrieved.

The patients included in the study underwent physical examination and Doppler sonographic evaluation. Prior to the examination the patients completed the Cold Intolerance Symptom Severity (CISS) scale, a 6-item questionnaire about symptoms of cold sensitivity in the treated hand, with a final score ranging from 4 (best) to 100 (worst); a score of 30 is considered as cutoff for cold intolerance [14]. Physical examination was performed by an orthopedic surgeon, independently of the treating hand surgeon, and included AED measurement of the affected finger and a neurovascular examination (including capillary refill time, two-point discrimination and Semmes-Weinstein monofilament).

Focused pulsed and power Doppler sonography were performed by two radiologists using a Siemens unit (ACUSSON 3000) equipped with a bandwidth seven to ten-megahertz linear array transducer. The transducer was held parallel to the long axis of the digital artery with minimal pressure [15]. Both the radial and ulnar digital artery were examined on the affected finger and the contralateral finger for comparison.

## Results

During the study period 1181 collagenase treatments were performed at the department. Three patients reported symptoms of possible vascular complications and participated in follow-up in October 2020 (Table 1). None of these patients reported any previous vascular symptoms in their hands prior to collagenase treatment.

**Table 1:** Patient characteristics and physical examination findings

	Patient 1	Patient 2	Patient 3
Age (years)	53	81	79
Sex	Female	Male	Female
Treated hand/finger	Right/5	Right/5	Left/5
AED baseline (°) MCP PIP	20 0	0 50	0 45
AED posttreatment (°) MCP PIP	0 0	0 30	0 0
AED follow-up (°) MCP PIP	0 0	0 30	0 30
Follow-up time (months)	82	72	17
Vascular complication onset (months)	3–5	3	6
CRF (s)	< 2	< 2	< 2
2-PD (mm)/SWMF	6/2.83	6/2.83	6/3.61
CISS	36	40	14

AED = active extension deficit; CISS = cold intolerance symptom severity; CRF = capillary refill time; MCP = metacarpophalangeal; PIP = proximal interphalangeal; SWMF = Semmes-Weinstein monofilament; 2-PD = 2-point discrimination

### Patient 1

A 53-year old right-handed non-smoking woman with a family history of Dupuytren disease presented in December 2013 with a primary contracture of the right small finger. She had no history of diabetes mellitus, vascular disease or previous hand trauma. A radial cord resulted in limited abduction of the small finger and an MCP joint contracture of 20° AED, with no contracture in the PIP joint. The patient was treated with collagenase injection, with subsequent manipulation two days later achieving complete correction without skin tears.

**Fig. 1:**
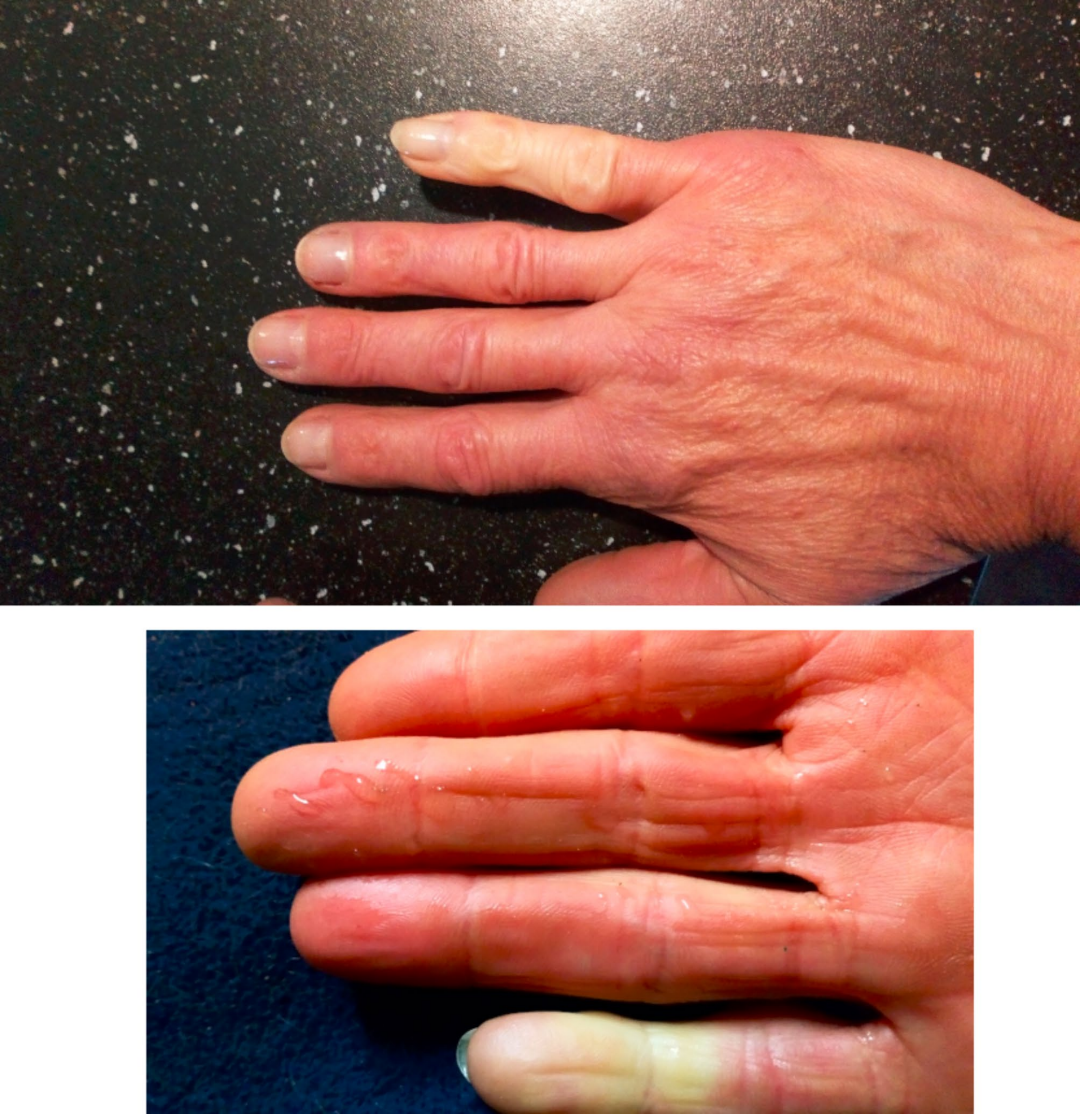
Episode of white discoloration of the right small finger in patient 1

A few months later during spring 2014 (3–5 months after treatment, could not be exactly specified by the patient) the patient experienced the first episode of white and bluish discoloration of the treated small finger (Fig. 1). The episodes became more frequent during autumn when the finger was exposed to cold temperatures, with almost daily episodes. The episodes subside within 30 min and are associated with mild to moderate pain, paresthesias, stiffness and weakness in the finger. The case was reported to the manufacturers Drug Safety department in 2016 as a possible treatment-related adverse event.

At follow-up in October 2020 physical examination revealed an AED of 0° in both the MCP and PIP joint. She has had no Dupuytren disease recurrence during the follow-up period and no further surgical procedures on the affected hand. Capillary refill time (< 2s), two-point discrimination (6 mm) and Semmes-Weinstein monofilament (2.83) were normal and similar to the contralateral side. The patient had a CISS score of 36 and reported no symptomatic improvement since onset. Doppler sonography revealed patent digital arteries in the right small finger.

### Patient 2

An 81-year old left-handed non-smoking man with a family history of Dupuytren disease presented in October 2014 with a recurrent contracture of the right small finger. He had previously undergone surgical fasciectomy twice (1997 and 2001). The patient had no history of diabetes mellitus or vascular disease but suffered from multiple comorbidities including polymyalgia rheumatica and a chronic knee arthroplasty infection. The patient presented with an isolated PIP joint contracture of 50° AED. At the extension procedure two days after collagenase injection the PIP joint was partially corrected to 30° AED, with no occurrence of skin tears.

In February 2015 (3 months after injection) the patient experienced and reported the first episode of white discoloration of the treated small finger when exposed to cold. Since then the patient has experienced daily episodes, associated with moderate pain, paresthesia and stiffness, relieved within minutes in warmer environment. The case was reported to the manufacturers Drug Safety department in 2016 as a possible treatment-related adverse event.

At follow-up in October 2020 physical examination revealed an isolated PIP joint contracture of 30° AED. No recurrence of contracture occurred during the follow-up period, but the patient had surgical excision of a painful palmar Dupuytren nodulus proximal to the ring finger in December 2016. Capillary refill time (< 2 s), two-point discrimination (6 mm) and Semmes-Weinstein monofilament (2.83) were normal and similar to the contralateral side. The patient had a CISS score of 40 and reported no symptomatic improvement since onset. Doppler sonography revealed no visible flow from the level of the PIP joint in the ulnar digital artery in the right small finger.

### Patient 3

A 79-year old right-handed non-smoking woman with a family history of Dupuytren disease presented in May 2019 with a primary contracture of the left small finger. The patient had treatment for hypothyroidism, but no history of diabetes mellitus, vascular disease or previous hand trauma. The patient had an isolated PIP joint contracture of 45° AED. The patient had a complete contracture correction after collagenase treatment without skin tear.

In December 2019 (6 months after injection), the patient reported the first episode of white discoloration of the treated small finger. The episode was not initiated by cold environment and was relieved within minutes. The patient had since then experienced four similar episodes with the last one occurring in June 2020. The episodes have been characterized by moderate paresthesias, but no pain, stiffness, or weakness.

At follow-up in October 2020 physical examination revealed a recurrent PIP joint contracture of 30° AED, with no subsequent procedures. Capillary refill time, two-point discrimination (6 mm) and Semmes-Weinstein monofilament (3.61) were normal and similar to the contralateral side. The patient reported a CISS score of 14, and improvement since she had no episodes over the last months. Doppler sonography revealed patent digital arteries in the left small finger.

## Discussion

The case series presented in this study further supports the evidence of the possibility of a persistent vascular complication after collagenase treatment for Dupuytren disease. It is however a very rare adverse event with an incidence of 0.25% in the study population, to compare with the incidence of neurovascular injuries after primary surgical fasciectomies of up to 5% [3, 6, 8]. In contrast, flexor tendon rupture, a well-known severe adverse event after collagenase injection, did not occur in any patient during the study period.

All patients in the study have in common that they were treated in the small finger, similar to the two previous case reports by Spiers et al. [10] and Kawano et al. [11]. A delayed presentation of a few months was observed in all cases, similar to the patient reported by Kawano et al. [11] (at 9 months), whereas the complaints started earlier in Spiers et al. [10[ case report (at 2 weeks). Two of the patients were treated for a PIP joint contracture, similar to the patient in the case report by Spiers et al. [10]. None of the patients in this case series or in the two previous case reports had severe contractures before treatment and only the patient reported by Spiers et al. [10] had sustained a minor skin tear after the finger extension procedure. No patient was an active smoker or had diabetes mellitus or preexisting vascular disease.

The cause of a vascular complication and reason to the delayed presentation is unknown. Although preclinical studies have demonstrated preservation of arterioles after local collagenase injection in an ex-vivo setting [4], an effect of collagenase on collagen in the vessel wall cannot be excluded. In this case the incidence may increase in patients treated with higher doses of collagenase, although this seems unlikely using our modified technique injecting multiple spots on the cord. Other possible explanations could be direct needle injury to the vessel, or injury related to the strain of the extension procedure or static nighttime splinting. This may lead to a small intimal tear in the arterial wall progressing over time to occlusive thrombus formation, intimal hyperplasia or fibrosis, which could explain the delayed onset of symptoms [16]. Patient 2 was the only patient in whom a nonpatent ulnar digital artery could be observed on the Doppler sonography distal to the PIP joint, similar to the patient in the case report by Spiers et al. [10]. Patient 2 had previously undergone surgical fasciectomy and it is possible that the artery could have sustained an injury during surgery, but he reported no vascular-related symptoms before the collagenase treatment. The other patients may have had a subclinical Raynaud phenomenon exacerbated by the treatment, similar to the case report by Kawano et al. [11]. Patient 3 reported spontaneous improvement at follow-up (no episodes during the last 5 months), suggesting potential reversibility which may be due to compensation from developed collateral circulation.

Adverse events after collagenase injection are well studied in both prospective studies and systematic reviews. Common adverse events, including skin tears, bruising, edema and lymphadenopathy, occur in the days after the injection and are usually transient and self-limiting requiring no further treatment [17, 18]. Severe adverse events, such as flexor tendon ruptures, are exceedingly rare [6]. Complications beyond the time of treatment are uncommon and the rarity of a vascular complication is supported by the absence of similar cases in studies with long-term follow up after collagenase treatment [9, 19, 20, 21, 22]. However, these studies might underestimate the incidence due to adverse events protocols not assessing potential vascular complications with a delayed onset. The risk of incidence underestimation is a limitation of our study. At the time of the delayed presentation no standardized follow-up of the patients was performed, and all patients in this study self-reported the symptoms. Strengths of the study includes prospectively collected baseline and posttreatment data of all patients treated with collagenase injection at the department. Furthermore, the study participants were thoroughly evaluated including baseline characteristics, physical examination, a relevant symptom validated patient reported outcome measure and Doppler sonography. Doppler sonography of the digital arteries is however an examiner-dependent examination. Our aim was to measure flow quality in digital arteries comparing with the contralateral side. Although we involved two radiologists performing multiple measurements, the results are difficult to interpret in terms of clinical relevance. Therefore, we have chosen only to assess the digital artery flow as patent or non-patent in this study.

## Conclusions

This study presents the first case series of patients with a delayed vascular complication after collagenase treatment for Dupuytren disease. Treating surgeons and patients should be aware of this rare complication, especially when treatment of the small finger is planned. Prior to treatment possible preexisting vascular symptoms should be inquired about and documented.

## Data Availability

All data generated or analysed during this study are included in this published article.

## Abbreviations

AED: Active extension deficit
CISS: Cold intolerance symptom severity
MCP: Metacarpophalangeal
PIP: Proximal interphalangeal
